# CD91 Derived Treg Epitope Modulates Regulatory T Lymphocyte Response, Regulates Expression of Costimulatory Molecules on Antigen-Presenting Cells, and Rescues Pregnancy in Mouse Pregnancy Loss Model

**DOI:** 10.3390/ijms22147296

**Published:** 2021-07-07

**Authors:** Anna Ewa Kedzierska, Daria Lorek, Anna Slawek, Tomasz Grabowski, Anna Chelmonska-Soyta

**Affiliations:** 1 Hirszfeld Institute of Immunology and Experimental Therapy, Polish Academy of Sciences, 53-114 Wroclaw, Poland; daria.lorek@hirszfeld.pl (D.L.); anna.slawek@hirszfeld.pl (A.S.); soyta@hirszfeld.pl (A.C.-S.); 2 Polpharma Biologics SA, Trzy Lipy 3, 80-172 Gdańsk, Poland; tomasz.grabowski@polpharmabiologics.com; 3 Department of Immunology, Pathophysiology and Veterinary Preventive Medicine, Wroclaw University of Environmental and Life Sciences, 50-375 Wroclaw, Poland

**Keywords:** tregs, bregs, pregnancy, epitope, CD80, CD86, antigen-presenting cells, miscarriage, MHCII, CD91

## Abstract

The loss of immune tolerance to fetal antigens may result in reproductive failure. The downregulated number and activity of T regulatory lymphocytes, which are critical for the establishment of immune tolerance to fetal antigens, during pregnancy may lead to miscarriage. The adoptive transfer of Tregs prevents fetal loss in abortion-prone mice. Recently, we demonstrated that the administration of tregitopes, which are short peptides found in human and mouse immunoglobulins (IgGs), decreased the incidence of abortions in female CBA/J mice mated with DBA/2J mice. Here, two non-IgG source peptides (SGS and LKD) that can potentially bind to the major histocompatibility complex II (MHC II) with high affinity and induce Treg expansion were designed in silico. The immune dysregulation-induced pregnancy failure mouse model was used to evaluate the effect of SGS and LKD on immune response and pregnancy outcome. The fetal death rate in the SGS-treated group was lower than that in the phosphate-buffered saline-treated group. SGS and LKD upregulated the splenic pool of Tregs and modulated the T-helper cell (Th1)/Th2-related cytokine response at the preimplantation stage. Additionally, SGS and LKD downregulated the expression of CD80 and MHC class II molecules in splenic CD11c^+^ antigen-presenting cells. Thus, SGS treatment can result in beneficial pregnancy outcomes. Additionally, SGS peptide-mediated immunomodulation can be a potential therapeutic strategy for immune dysregulation-induced pregnancy failure.

## 1. Introduction

The cross-talk between the mother and the conceptus is one of the fundamental prerequisites for the development of a healthy pregnancy. The recognition of foreign paternal antigens in the semen as well as in the embryo promotes immune tolerance to fetal antigens and enables embryo implantation and fetal development. The mechanisms underlying natural tolerance during pregnancy have not been elucidated. However, dysregulated immune tolerance during pregnancy is reported to promote various reproductive complications such as preeclampsia or spontaneous and recurrent miscarriage. Miscarriages affect one in four diagnosed pregnancies, and about half of them do not have determined etiology. It is believed that women with recurrent miscarriages have impaired tolerance to autoantigens and fetal antigens [1]. It is well known that regulatory T lymphocytes (Tregs) and regulatory B lymphocytes (Bregs) contribute to the development of tolerance to fetal antigens [2,3]. In both humans and mice, the proportion of Tregs in pregnant individuals is higher than that of those in non-pregnant controls. However, the proportion of Tregs in spontaneous abortion cases is lower than that of healthy pregnancy cases but higher than that of non-pregnant cases [4,5,6,7,8,9,10]. Similarly, compared to non-pregnant subjects, the proportion of Bregs is higher in pregnancy cases. Additionally, the proportion of Bregs in spontaneous abortion cases is lower than it is in healthy pregnancy cases [11,12,13]. The abortion-prone model (CBA/J female mice mated with DBA/2J males), which is a widely studied murine model of immune-mediated pregnancy failure, is characterized by a high rate of fetal resorption and mortality [14]. Previous studies have demonstrated that the adoptive transfer of regulatory B and/or T cells can reverse the high abortion rate in abortion-prone mice if the cells are administered immediately after fertilization [12,15]. Recently, we demonstrated that the early administration of T regulatory cell epitopes (tregitopes), which are short peptides found in the light and heavy chains of human and mouse immunoglobulins (IgGs), decreased the incidence of abortion in abortion-prone mice [16]. These tregitopes may bind to the major histocompatibility complex (MHC II)-binding groove, and this complex is presented to Tregs, which results in the activation and proliferation of Tregs. IgG-derived tregitopes, identified by de Groot and coworkers, are formed after IgG is internalized and processed by the antigen-presenting cells (APCs) [17]. However, the sequences of putative tregitopes have also been reported in other self-proteins, such as albumin, fibrinogen, and osteocalcin [17,18]. Moreover, viruses, fungi, and bacteria are reported to express tolerogenic proteins [19,20,21,22], which may be the source of putative tregitope sequences. Therefore, this study aimed to identify novel non-IgG source peptides that can bind to MHC II with high affinity based on an in silico model and to investigate whether their early administration promotes the expansion of regulatory lymphocytes and prevent abortion in a mouse abortion-prone model. The findings of this study suggested that the early administration of CD91 derived Treg epitope (SGS peptide) induces the expansion of Tregs and may prevent abortions in an abortion-prone mouse model. The identified SGS peptide meets the criteria of a tregitope, as its sequence is present in prevalent human proteins, conservative across many species, and can bind to the human leukocyte antigen-DR isotype (HLA-DR) [23].

## 2. Results

### 2.1. Peptides Selection

Here, two peptides, the sequences of which were identified in the proteins involved in the immune system were chosen (Table 1). The sequence of the first synthetic peptide was LKDFALEGTLAADKT (LKD). The calculated half-maximal inhibitory concentration value (IC_50_) of LKD against the MHC II molecule was 108 nM. The LKD peptide was chosen as a control peptide because we have previously shown that it did not induce Tregs expansion in vitro [24] and could not be treated as a tregitope. The sequence of the second peptide was SGSVVLRNSTTLVMH (SGS). The IC_50_ of SGS against MHC II was 146 nM. The peptides were commercially synthesized, and the sequence purity was above 90% (GeneCust, Luxembourg).

### 2.2. SGS Peptide Decreases the Abortion Rate

The protective role of SGS and LKD during pregnancy in abortion-prone mice was examined by analyzing the number of non-resorbed and resorbed embryos. The fetal death rate at 14 dpc was calculated according to the formula described in the methods section.

**Figure 1:**
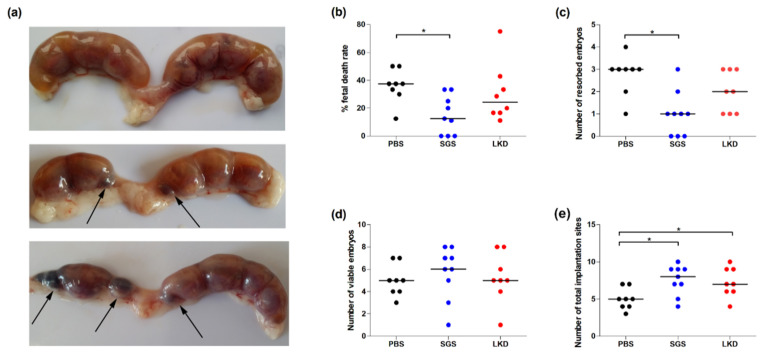
(**a**) Representative pictures of the uteri showing resorbing (arrows) and viable implantation sites and the effect of SGS and LKD peptides on the (**b**) fetal death rate, (**c**) number of resorbed embryos, (**d**) number of viable embryos, and (**e**) total implantation sites at 14 dpc in abortion-prone mice. The data were analyzed using one-way analysis of variance (normally distributed data), followed by Bonferroni’s multiple comparison post hoc test or Kruskal–Wallis test (non-normal distributed data), followed by Dunn’s multiple comparison post hoc test (*p* < 0.05). Data are presented as individual values with the median. * *p* < 0.05.

The fetal death rate (15.03%) and the number of resorbed embryos in the SGS-treated group were significantly lower than those in the PBS-treated group (36.04%) (Figure 1b,c) (*p* < 0.05). The number of viable embryos in the SGS-treated and LKD-treated groups was not significantly (*p* > 0.05) different from that of the PBS-treated group (Figure 1d). However, the total number of implantation sites in the LKD-treated and SGS-treated group was significantly (*p* < 0.05) higher than that of the PBS-treated group (Figure 1e).

### 2.3. Designed Peptides Enhance Serum Interleukin 10

**Figure 2:**
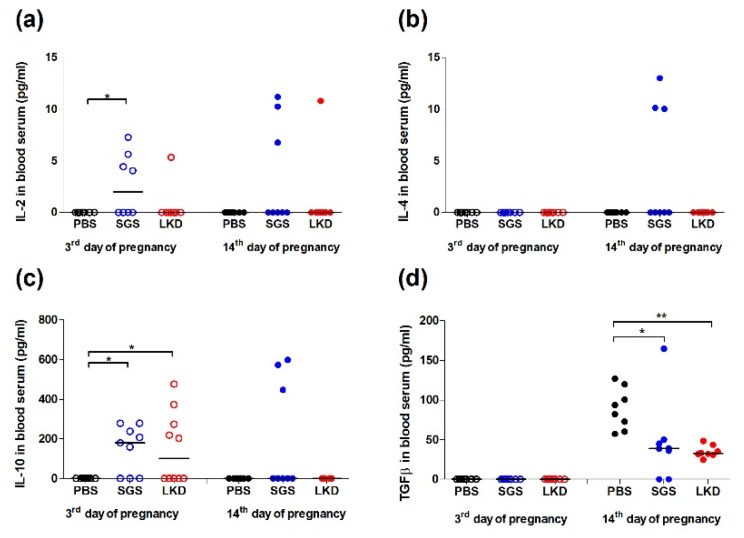
Effect of SGS and LKD on the levels of (**a**) IL-2, (**b**) IL-4, (**c**) IL-10, and (**d**) TGFβ1 at 3 dpc and 14 dpc in abortion-prone mice. Data were analyzed using one-way analysis of variance (for normally distributed data), followed by Bonferroni’s multiple comparison post hoc test or the Kruskal–Wallis test (for non-normally distributed data), followed by Dunn’s multiple comparison post hoc test (*p* < 0.05). Data are presented as individual values with the median. * *p* < 0.05 and ** *p* < 0.01.

The effect of SGS and LKD on the T-helper cell (Th1)/T-helper cell (Th2) balance during pregnancy was examined in abortion-prone mice by measuring the serum levels of interleukin 2 (IL-2), interleukin 4 (IL-4), interleukin 10 (IL-10), transforming growth factor-beta 1 (TGFβ1), and interferon-gamma (IFNγ). At 3 dpc, the serum level of IL-2 was upregulated in the SGS-treated group (Figure 2a). However, the IL-2 serum levels were not significantly (*p* > 0.05) different between the experimental groups at 14 dpc (Figure 2a).

The serum levels of IL-4 on the third and fourteenth days of pregnancy were not significantly different (*p* > 0.05) between the experimental groups (Figure 2b). Compared to those in the PBS-treated group, the IL-10 serum levels on the third day of pregnancy were significantly upregulated in the SGS-treated and LKD-treated groups (*p* < 0.05) (Figure 2c). At 14 dpc, the serum levels of IL-10 were not significantly different between the experimental groups. On the third day of pregnancy, the TGFβ1 serum levels were not significantly different between the experimental groups (Figure 2d). However, the TGFβ1 serum levels in the SGS-treated and LKD-treated groups were significantly lower than those in the PBS-treated group at 14 dpc (Figure 2d; *p* < 0.05). The absorbance values of IFNγ on the third and fourteenth day of pregnancy were below the detection level of the kit (data not shown).

### 2.4. SGS and LKD Peptides Upregulate Treg Frequency

**Figure 3:**
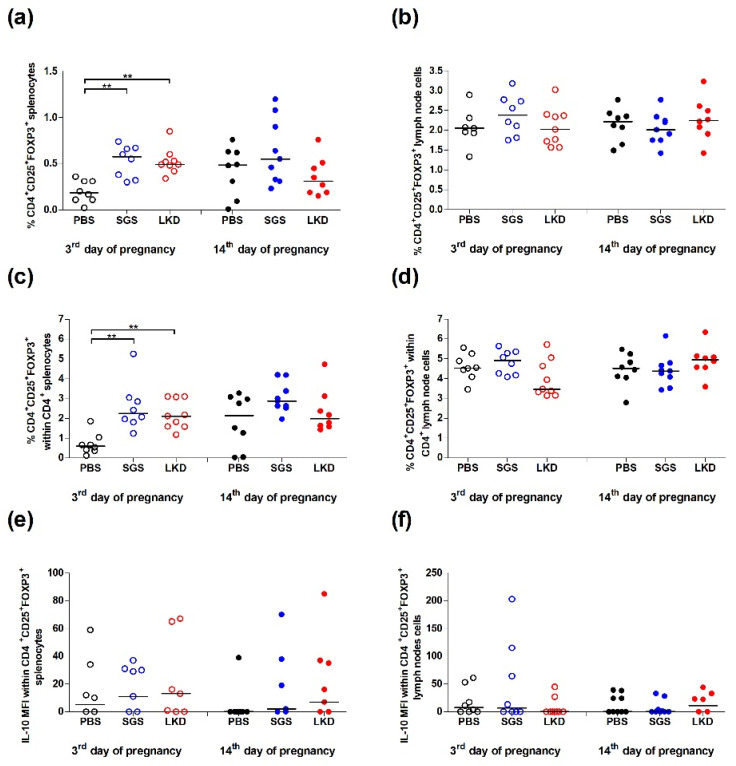
Frequencies of CD4^+^CD25^+^FOXP3^+^ (**a**) splenocytes and (**b**) uterine-draining lymph node cells among the (**c**) CD4^+^ splenocytes and (**d**) CD4^+^ lymph node cells, respectively, of the SGS-treated and LKD-treated groups at 3 dpc and 14 dpc in abortion-prone mice. The median fluorescence intensity (MFI) of IL-10 in the CD4^+^CD25^+^FOXP3^+^ (**e**) splenocytes and (**f**) uterine-draining lymph node cells of the SGS-treated and LKD-treated groups at 3 dpc and 14 dpc in abortion-prone mice. Data were analyzed using one-way analysis of variance (for normally distributed data), followed by Bonferroni’s multiple comparison post hoc test or the Kruskal–Wallis test (for non-normally distributed data), followed by Dunn’s multiple comparison post hoc test (*p* < 0.05). Data are presented as individual values with the median. ** *p* < 0.01.

**Figure 4:**
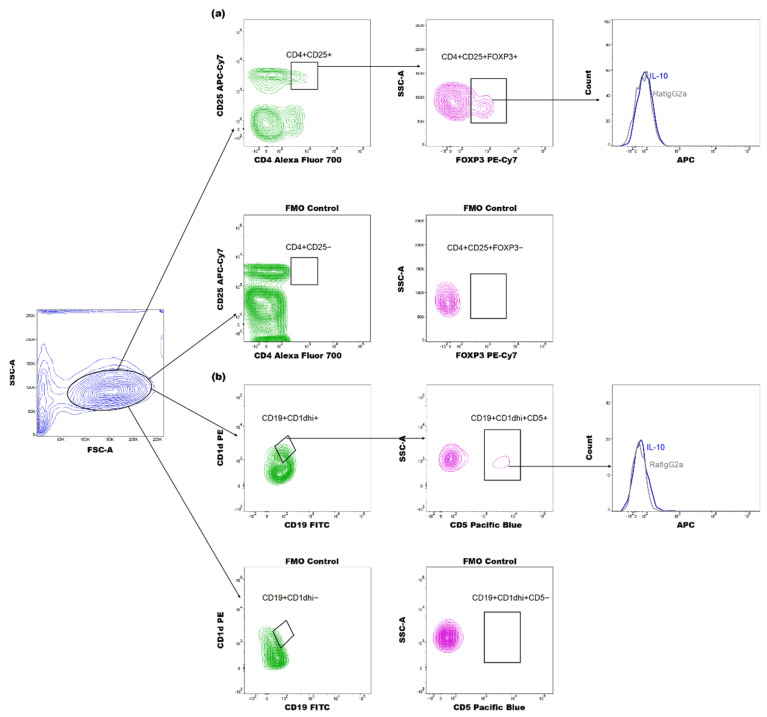
Representative dot plots for gating regulatory T (Tregs) and B lymphocytes (Bregs) and representative histograms of the expression of IL-10 (blue histograms) overlayed with those in the respective isotype-matched controls (red histograms) derived from the spleens of abortion-prone mice. (**a**) Gating strategy for CD4^+^CD25^+^FOXP3^+^ lymphocytes and IL-10 expression in these cells. (**b**) CD19^+^CD1d^high+^CD5^+^ lymphocytes and expression of IL-10 in these cells.

Next, the effect of SGS and LKD on Treg expansion was examined in abortion-prone mice. The number of CD4^+^CD25^+^FOXP3^+^ lymphocytes in the spleen and para-aortic uterine-draining lymph nodes (PALNs) (Figure 3) of the pregnant mice were analyzed. The gating strategy for analyzing Tregs is shown in Figure 4.

At 3 dpc, the frequencies of splenic CD4^+^CD25^+^FOXP3^+^ cells in the SGS-treated and LKD-treated groups were almost two times higher (*p* < 0.01) than those in the PBS-treated group (Figure 3a). The frequencies of CD4^+^CD25^+^FOXP3^+^ cells in the PALN were similar among the experimental groups (Figure 3b). The proportion of CD4^+^CD25^+^FOXP3^+^ cells was upregulated in the CD4^+^ splenocyte population of the SGS-treated and LKD-treated groups (Figure 3c). The frequency of Tregs among the CD4^+^ PALNs was similar among the experimental groups (Figure 3d). Furthermore, the levels of IL-10 (calculated as MFI) in the splenic and lymph node Tregs were similar between the SGS-treated, LKD-treated, and PBS-treated groups (Figure 3e,f).

The administration of SGS and LKD did not affect the frequency of Tregs in the spleen and PALN at 14 dpc (Figure 3a,b). Moreover, the proportion of CD4^+^CD25^+^FOXP3^+^ cells within CD4^+^ cells in the spleen and PALN of the SGS-treated and LKD-treated groups was similar between the experimental groups (Figure 3c,d). Additionally, the IL-10 levels in the splenic and lymph node Tregs (*p* > 0.05) were similar among the experimental groups (Figure 3e,f).

### 2.5. LKD Upregulates Breg Frequency

**Figure 5:**
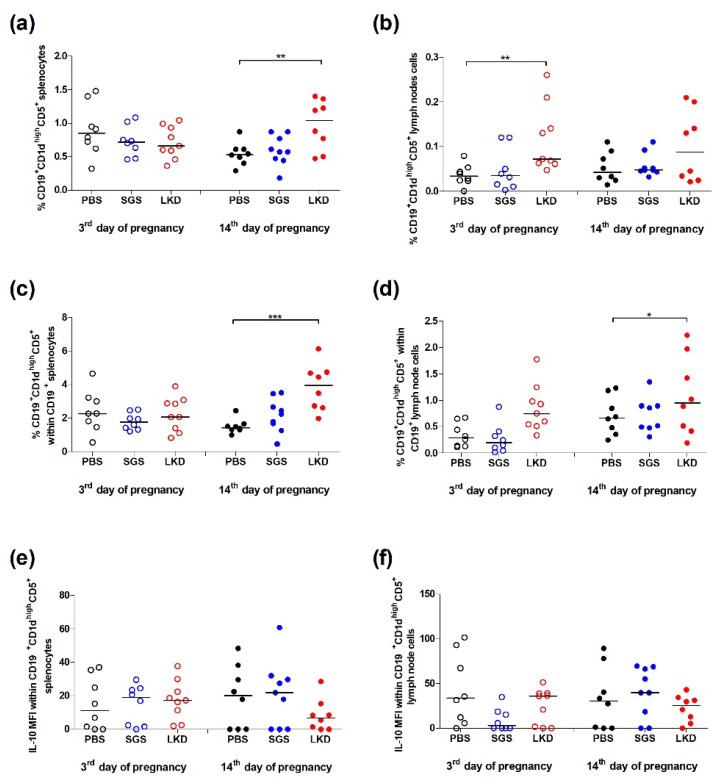
Effect of SGS and LKD on the proportions of CD19^+^CD1d^+^CD5^+^ (**a**) splenocytes and (**b**) uterine-draining lymph node cells in the (**c**) CD19^+^ splenocytes and (**d**) CD19^+^ uterine-draining lymph nodes, respectively, and the production of IL-10 by the CD19^+^CD1d^+^CD5^+^ (**e**) splenocytes and (**f**) lymph node cells at 3 dpc and 14 dpc in abortion-prone mice. Data were analyzed using one-way analysis of variance (for normally distributed data), followed by Bonferroni’s multiple comparison post hoc test or the Kruskal–Wallis test (for non-normal distributed data), followed by Dunn’s multiple comparison post hoc test (*p* < 0.05). Data are presented as individual values with the median. * *p* < 0.05, ** *p* < 0.01, and *** *p* < 0.001.

The effect of SGS and LKD on the expansion of Bregs was examined in abortion-prone mice. The proportion of CD19^+^CD5^+^CD1d^hi^ cells (Bregs) in the spleen and PALN was examined (Figure 5) based on the gating strategy shown in Figure 4b.

The frequency of Bregs in the spleens and among B cell populations (Figure 5a,c, respectively) was similar among the experimental groups on the third day of pregnancy (*p* > 0.05). However, at 3 dpc, the proportion of Bregs (*p* < 0.01) in the PALN (*p* < 0.01) in the LKD-treated group was higher than that of the PBS-treated group (Figure 5b). The frequency of Bregs among B cell populations in the PALN in the SGS-treated and LKD-treated groups was not significantly different from that of the PBS-treated group (*p* > 0.05) (Figure 5d). The IL-10 levels in the Bregs cells were not markedly different in the spleen and PALN at 3 dpc (Figure 5e,f).

On the fourteenth day of pregnancy, the frequency of Bregs in the spleen (*p* < 0.01) and among splenic B cell populations (*p* < 0.001) in the LKD-treated group was higher than that of the PBS-treated group (Figure 5a,c). However, at 14 dpc, the proportion of Bregs in the PALN in the SGS-treated and LKD-treated groups was not significantly different from that of the PBS-treated group (*p* > 0.05) (Figure 5b). However, the frequency of Bregs among B cell populations in the PALN in the LKD-treated groups was not significantly different from that in the PBS-treated group (*p* > 0.05) (Figure 5d). Additionally, the level of IL-10 in the splenic and lymph node Tregs in the SGS-treated and LKD-treated groups was similar to that of the PBS-treated group (Figure 5e,f).

### 2.6. SGS and LKD Peptides Modulate the Costimulatory Phenotype of APCs

**Figure 6:**
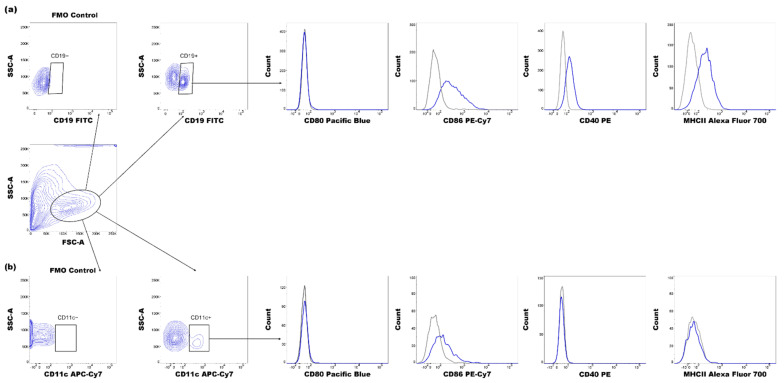
Representative histograms of the expression of CD40, CD80, CD86, and major histocompatibility class II molecules (blue histograms) overlayed with those in the respective isotype-matched controls (gray histograms) in (**a**) CD19^+^ cells and (**b**) CD11c^+^ cells derived from the spleens of abortion-prone mice.

Next, the effect of SGS and LKD on the costimulatory phenotype of APCs was examined. The expression of CD40, CD80, CD86, and MHC class II molecules on the surface of DCs (CD11c^+^) and B lymphocytes (CD19^+^) was analyzed based on the gating strategy shown in Figure 6.

**Figure 7:**
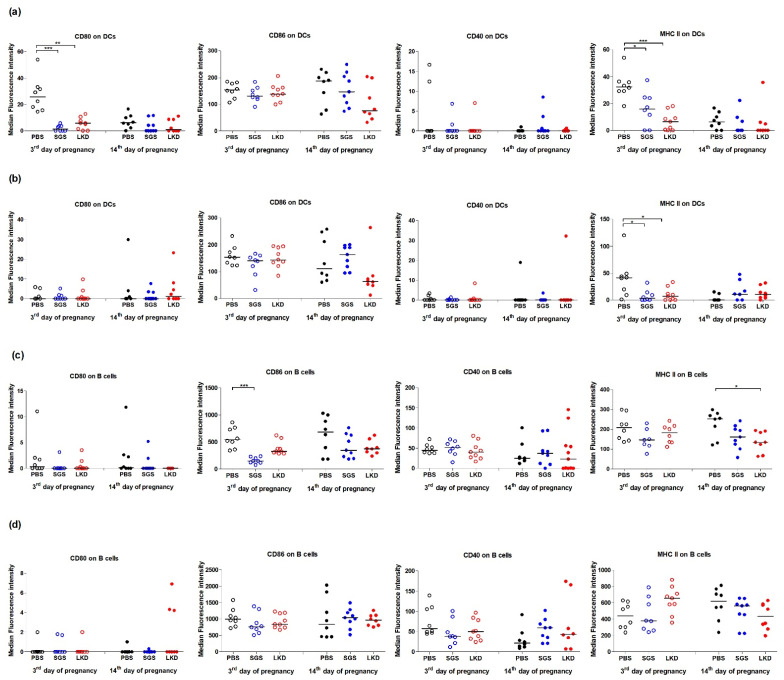
Effect of SGS and LKD on the expression of CD40, CD80, CD86, and major histocompatibility class II proteins on the surface of (**a**) CD11c^+^ splenocytes and (**b**) CD11c^+^ uterine-draining lymph node cells, (**c**) CD19^+^ splenocytes, and (**d**) CD19^+^ uterine-draining lymph node cells at 3 dpc and 14 dpc in abortion-prone mice. Data were analyzed using one-way analysis of variance (for normally distributed data), followed by Bonferroni’s multiple comparison post hoc test or the Kruskal–Wallis test (for non-normally distributed data), followed by Dunn’s multiple comparison post hoc test (*p* < 0.05). Data are presented as individual values with the median. * *p* < 0.05, ** *p* < 0.01, and *** *p* < 0.001.

On the third day of pregnancy, the expression levels of CD80 on the splenic CD11c^+^ cells in the SGS-treated and LKD-treated groups (*p* < 0.01, LKD peptide; *p* < 0.001 for SGS peptide) were significantly downregulated when compared with those in the PBS-treated group (Figure 7a). However, the expression levels of CD80 on the CD11c^+^ splenic cells in the PALN were similar between the SGS-treated, LKD-treated, and PBS-treated groups (Figure 7b). The expression levels of CD86 and CD40 on the DCs (*p* > 0.05) in the spleen and PALN were not significantly different between the experimental groups (Figure 7a,b). Meanwhile, the expression levels of MHC II molecules on the DCs in the spleen and PALN of the SGS-treated and LKD-treated groups were downregulated when compared to those in the spleen and PALN of the PBS-treated group (Figure 7a,b). SGS and LKD did not affect the expression of CD80, CD40, and MHC II on the B cells in the PALN and spleen (Figure 7c,d). However, treatment with SGS inhibited CD86 expression on B cells in the spleen (*p* < 0.001) but not on those in the PALN (Figure 7c,d).

On the fourteenth day of pregnancy, SGS and LKD did not upregulate the expression of CD80, CD86, and CD40 molecules on DCs or B cells in the spleen and PALN (*p* > 0.05). The expression of MHC II molecules on the splenic B cells, but not on splenic DCs, was downregulated in the LKD-treated group when compared to that of the PBS-treated group. However, the expression of MHC II molecules in the PALN was similar between the SGS-treated, LKD-treated, and PBS-treated groups (Figure 7c,d).

## 3. Discussion

Dysregulated immune tolerance to fetal antigens may cause various reproductive complications. The decreased number of Tregs, which are critical for the establishment of immune tolerance to fetal antigens, during pregnancy may result in miscarriage. In this study, the mouse model of immune imbalance-mediated pregnancy failure was used to study the effect of newly designed peptides on pregnancy outcomes.

Tregitopes, which are short amino acid sequences found in the light and heavy chains of human and mouse IgG, can bind to MHC II molecules with high affinity. Additionally, tregitopes presented with MHC II promote the expansion of Tregs and suppress the effector T cell responses by enhancing the production of cytokines [17]. Based on a previously reported in silico model [24], two potential non-IgG source Treg epitopes that can induce the expansion of Tregs were identified in this study. The main assumption of the in silico analysis in this study was that the designed tregitopes can bind to MHC II molecules, which is an essential attribute for presenting tregitopes. However, binding with a high affinity to MHC II does not always result in the induction of Tregs. To ensure that the selected tregitopes exhibit immunomodulatory features, other factors, such as hydrophobicity index, the number of negatively and positively charged residues, and immune function, were incorporated in the analysis [24].

In this study, the number of resorbed and viable fetuses was examined to analyze the effect of SGS and LKD on pregnancy outcomes. The fetal death rate and the number of resorbed embryos in the SGS-treated group were significantly lower than those of the PBS-treated group. Additionally, treatment with SGS increased the number of implantation sites, which suggested that SGS can potentially rescue pregnancy in abortion-prone mice. Previously, we had demonstrated that known tregitopes decreased the occurrence of abortion in abortion-prone mice [16]. SGS may rescue pregnancy in abortion-prone mice by promoting the expansion of Tregs and regulating the levels of serum cytokines.

The frequency of Tregs during a healthy pregnancy is reported to be higher than during a non-pregnancy period. Additionally, the frequency of Tregs in abortion cases is lower than that in healthy pregnancy cases [7,8]. Compared to those in mice with healthy pregnancies, the local and peripheral levels of Tregs in the uterus are downregulated during pregnancy in abortion-prone mice [27]. The adoptive transfer of splenic and thymic CD4^+^CD25^+^ cells from CBA/J mice mated with BALB/c males (healthy pregnancy) into abortion-prone mice inhibited the incidence of abortion [27]. In this study, SGS and LKD promoted peripheral changes in the frequency of Tregs at the preimplantation stage of pregnancy. The SGS-induced or LKD-induced expansion of Tregs is consistent with the results of our previous studies, which reported that the administration of known tregitopes promoted the induction of splenic Tregs [16]. However, the results of our previous study indicated that tregitopes increased the level of IL-10 produced by Tregs, which was not observed in this study. The IL-10 serum levels were upregulated at the preimplantation stage of pregnancy in the SGS-treated and LKD-treated groups. Cytokines are essential factors for the survival of the fetus [28,29,30,31,32,33]. The abortion-prone mouse model exhibits a dysregulated cytokine balance and downregulated levels of IL-10 and IL-4 in feto-placental units [34]. The administration of exogenous IL-10 prevents fetal loss in abortion-prone mice, whereas the depletion of IL-10 promotes abortion [34]. This indicates that SGS protects against fetal loss through the upregulation of the IL-10 level. Like IL-10, TGFβ is one of the cytokines that is crucial in the modulation of the dendritic cells phenotype and the differentiation of Tregs [35,36]. It is well known that TGFβ may inhibit the proliferation and differentiation of mature Treg. Here, we demonstrated an increased level of IL-10 at 3 dpc and a decreased TGFβ1 level at 14 dpc. The upregulation of IL-10 may be related to the observed expansion of Tregs at 3 dpc. However, the lack of differences in Tregs expansion at 14 dpc suggests that the downregulation of TGFβ1 did not negatively influence immune tolerance. This conclusion may be supported by the fact that, despite decreased TGFβ1 concentration, the SGS-treated mice had a reduced fetal death rate. However, this is very speculative, and additional studies are needed to explain this phenomenon. Additionally, SGS upregulated the IL-2 level at the preimplantation stage of pregnancy. The cytokine microenvironment plays a key role in the differentiation, expansion, and function of Tregs. IL-2 is one of the cytokines essential for the development, peripheral homeostasis, and stability of Tregs [37]. Therefore, the effectiveness of SGS can be also attributed to its ability to upregulate IL-2 levels. The protective role of the SGS peptide on pregnancy outcome may be due to its origin. The sequence of the SGS peptide was identified in an extracellular α-chain with binding sites for ligands of prolow-density lipoprotein receptor-related protein 1 isoform X1 from various species, such as *Mus musculus* and *Homo sapiens*. The prolow-density lipoprotein receptor-related protein 1 (LRP1), also known as the CD91 molecule, is an endocytic receptor that recognizes over thirty different ligands, which play various roles in many biological processes such as cell signaling, the activation of lysosomal enzymes, lipoprotein metabolism, phagocytosis, and endocytosis [25]. CD91 is expressed by most APCs and participates in the regulation of inflammatory processes and cross-presentation of the heat shock proteins (HSPs)-chaperoned peptide [38]. Through metalloproteinases, a shed form of LRP1 may be released into the extracellular matrix in response to stress and inflammatory mediators and to function as a decoy receptor. LRP1/CD91 shedding results in the release of ligand-binding α-chain from the cell [39]. The released soluble LRP1 (sLRP1) can be detected in the brain, plasma, cerebrospinal fluid, and the peripheral nervous system [39,40,41]. During the incubation of human plasma sLRP1, purified mouse LRP1 with RA 264.7 macrophage-like cells increased mRNA for IL-10 [42]. The exact mechanism associated with the biological activity and immune functions of LRP1/CD91 remains unresolved. However, we believe that the delivered amount of SGS peptide might induce, as observed in this study, increased concentration of the IL-10 in blood sera, which in turn might prompt Tregs and contribute to improved pregnancy outcomes in abortion-prone mice.

Although the LKD peptide could not rescue pregnancy, it increased the number of CD19^+^CD5^+^CD1d^high+^ cells (Bregs) in the lymph nodes (at the preimplantation stage) and spleen at 14 dpc, which was not observed after treatment with the SGS peptide. Moreover, LKD administration increased the serum level of IL-10 but did not upregulate the levels of IL-10 (calculated as MFI) in the splenic and lymph node Bregs. It was already demonstrated that the proportion of Bregs, called B10 cells in abortion-prone mice, is lower than that of mice with healthy pregnancies. Moreover, the adoptive transfer of IL-10-producing B cells mitigated the incidence of abortion in abortion-prone mice [11]. It was also demonstrated that IL-10 secreting B cells (B10), but not B effector cells that do not produce IL-10, are prominent in the maintenance of the immune balance during pregnancy [43]. Furthermore, in our previous work, we have shown that at 14 dpc, abortion-prone mice have an increased proportion of CD19^+^CD5^+^CD1d^high+^ cells in the uterine draining lymph nodes when compared to that of mice with healthy pregnancies [44]. What is more, the frequency of CD19^+^CD5^+^ cells in preeclampsia cases is higher than that of the second and third trimester of healthy pregnancy cases and is correlated with the production of autoantibodies. The autoantibodies generated from the CD19^+^CD5^+^ cells may reach the placenta and consequently lead to pregnancy failure [45]. Therefore, B cells are reported to exert both beneficial and adverse effects on pregnancy. These contradictory findings can be attributed to the complicated biology of B cells and the differential roles of B cell subpopulations in adaptive and innate immunity [3,46,47]. Thus, we hypothesized that the elevated frequencies of CD19^+^CD5^+^CD1d^high+^ cells after LKD treatment may have an adverse effect on pregnancy outcome.

B cells and DCs can present antigens to the T lymphocytes through MHC II molecules. Antigen presentation in the absence of CD80/CD86 costimulatory molecules and cytokines, such as IL-10, induces the differentiation of T cells into Tregs. Previously, we demonstrated that the early administration of known IgG-derived epitopes (mouse tregitopes 289 and 167) downregulated the expression of CD80 and CD86 antigens on DCs and B cells at 3 dpc and 14 dpc [16]. In this study, SGS and LKD downregulated the expression of CD80 and MHC II molecules on splenic DCs. Additionally, SGS and LKD downregulated the expression of MHC II on DCs residing in the lymph nodes at the preimplantation stage of pregnancy. Among the costimulatory molecules on B cells, SGS downregulated the expression of the CD80 antigen and consequently inhibited maternal rejection of fetuses in abortion-prone mice. The downregulated expression of costimulatory molecules on DCs promotes immune tolerance to self-antigens. Similar observations were reported for B cells in a previous study, which demonstrated that the inhibition of CD86 induced antigen-specific peripheral tolerance [48]. Muzzio et al. (2014) demonstrated that the proportions of peritoneal CD19^+^CD23^−^CD5^+^CD86^+^ B-1a B cells in mice with healthy pregnancy was significantly lower than that of animals associated with fetal loss or non-pregnant animals [49]. Previously, we had reported that the mRNA expression of CD86 in the splenic B cells of mice with healthy pregnancy is downregulated when compared to that in the splenic B cells of mice with disturbed pregnancy [50]. Furthermore, the systemic inhibition of CD80 and CD86 at the implantation period of pregnancy inhibited [51,52] the rejection of fetuses in abortion-prone mice. Therefore, we hypothesized that the SGS-mediated inhibition of costimulatory molecules and MHC II on APCs may exert beneficial effects on fetal development by inducing tolerance to embryo antigens. SGS-mediated downregulation of CD80, CD86, and MHC II on APCs and the expansion of Tregs are consistent with the findings of de Groot et al., who reported that treatment with known tregitopes downregulated the expression of costimulatory molecules [53].

In summary, this study demonstrated that treatment with the novel CD91 derived peptide (SGS) with tregitope activity increased the proportions of Tregs, upregulated the concentrations of IL-2 and IL-10, and downregulated the expression of the costimulatory molecules of APCs, which resulted in improved pregnancy outcomes. The findings of this study indicate that the SGS peptide may mimic the pro-tolerogenic effects of known tregitopes and that SGS is a potential therapeutic for immune dysregulation-induced pregnancy failure. However, further studies are needed to evaluate the ability of SGS to regulate the immune response in humans. Although LKD promoted the expansion of Tregs and Bregs, it did not decrease the incidence of abortions, which may be due to its unverified tregitope activity [24] and therefore its insufficient immunomodulatory effects.

## 4. Materials and Methods

### 4.1. Peptides Selection and Design

The tregitopes were designed as described previously [24]. First, tregitopes were generated using an in silico mathematical model for the correlation between the chemical structure of tregitopes and binding strength with MHC II (DRB1*04:01). In this study, the following parameters were calculated for the tregitope sequences: the half-maximal inhibitory concentration value against MHC II, the hydrophobicity index, the number of negatively and positively charged residues, the aliphatic index, the isoelectric point, the hydrogen bond donors, and the topological polar surface area. In silico analysis of 16 amino acid sequences of IgG with tolerogenic function similar to that of tregitopes was performed for mathematical selection. The linear validated correlation between binding strength with MHC II and physicochemical properties has been suggested instead of the present epitope cluster classification (unpublished data).

### 4.2. Animals

DBA/2J male mice and CBA/J female mice, which were purchased from Charles River Laboratories (Sulzfeld, Germany), were housed under specific pathogen-free conditions with a 12 h dark/light cycle. The vaginal swabs were collected from female CBA/J mice aged 6–8 weeks every morning to determine the phase of the reproductive cycle. Next, the vaginal smears were fixed and stained using the Cytocolor reagent (Merck Millipore, Burlington, Massachusetts, USA), as per the manufacturer’s instructions. Female mice at the proestrus phase were mated with DBA/2J males in the evening (at 7 pm) on the same day. Copulation was confirmed the next morning based on the presence of a vaginal plug. Pregnancy was defined as 0 days post coitum (dpc). At 0 dpc, the female mice were intraperitoneally injected with 100 µg (dissolved in 150 µL phosphate-buffered saline (PBS)) SGS peptide (SGS-treated group, n = 8 at 3 dpc and n = 9 at 14 dpc), 100 µg LKD peptide (LKD-treated group, n = 9 at 3 dpc and n = 8 at 14 dpc), or 150 µL PBS (PBS-treated group, n = 8). At 3 and 14 dpc, the blood samples, para-aortic uterine-draining lymph nodes (PALNs), and uteri were collected for further analyses. All efforts were made to minimize animal suffering. The animal experiments were approved by the Local Ethics Committee for Experiments on Animals at the Hirszfeld Institute of Immunology and Experimental Therapy in Wroclaw (No. 53/2015).

### 4.3. Tissue Processing

The spleen and PALN were isolated as described previously [16]. First, the spleen and PALN were passed through a 40-μm cell strainer (Falcon) into lysis solution (0.84% ammonium chloride) and wash buffer (PBS supplemented with 2 mM ethylenediaminetetraacetic acid (EDTA) and 2% fetal bovine serum (FBS; Biowest, France)), respectively. The cells were washed twice (at 4 °C and 300× *g* for 10 min) with wash buffer and stimulated as described in the flow cytometry section.

The uterus was collected from pregnant females at 14 dpc to determine the fetal death rate, which was calculated as follows: [(number of resorbed embryos)/(resorbed embryos + viable embryos)] × 100. The abortion sites were identified (Figure 2a) as described previously [16].

### 4.4. Flow Cytometry

The cells (1 × 10^6^ cells) were stimulated as previously described [16]. First, the cells were incubated with 1 µg/mL ionomycin (Cayman Chemical, Ann Arbor, MI, USA), 0.1 µg/mL phorbol 12-myristate 13-acetate (Cayman Chemical, Ann Arbor, MI, USA), 2 µM monensin (eBioscience, Waltham, MA, USA), and 10 µg/mL brefeldin A (eBioscience, Waltham, MA, USA) in Rosewell Park Memorial Institute-1640 medium supplemented with 10% FBS and 1X penicillin/streptomycin (from 100X) (Merck Millipore, Burlington, MA, USA) at 37 °C and 5% CO_2_ for 6 h. The cells were then stained with allophycocyanin (APC)/Cy7-conjugated anti-mouse CD25 (BD Biosciences; clone: PC61), Alexa Fluor 700-conjugated anti-CD4 (eBioscience, USA; clone: Gk1.5), phycoerythrin (PE)-conjugated anti-CD1d (eBioscience, USA; clone: 1B1), fluorescein isothiocyanate (FITC)-conjugated anti-CD19 (eBioscience, USA; clone: eBio1D3), pacific blue-conjugated anti-CD5 (eBioscience, USA; clone: 53-7.3), FITC-conjugated anti-CD19 (eBioscience, USA; clone: eBio1D3), APC/Cy7-conjugated anti-CD11c (eBioscience, USA; clone: N418), PE/Cy7-conjugated anti-CD86 (eBioscience, USA; clone: GL1), pacific blue-conjugated anti-CD80 (eBioscience, USA; clone: 16-10A1), Alexa Fluor 700-conjugated anti-MHC class II (IA/IE) (eBioscience, USA; clone: M5/114.15.2), PE-conjugated anti-CD40 (eBioscience, USA; clone: 1C10) antibodies, or isotype controls for 30 min at 4 °C in the dark. Next, the cells were washed and fixed with fixation/permeabilization buffer (eBioscience, USA) for 14 h at 4 °C in the dark. The cells were then washed twice with permeabilization buffer (eBioscience, USA) and blocked with anti-CD16/CD32 antibodies for 15 min at 4 °C in the dark. The cells were then stained with APC-conjugated anti-mouse interleukin 10 (IL-10) (eBioscience, USA; clone: JES5-16E3) and PE/Cy7-conjugated anti-Foxp3 antibodies (eBioscience, USA, clone: FjK-16s) or isotype controls for 1 h at 4 °C in the dark. The cells were washed twice with permeabilization buffer (eBioscience, USA), and the fluorescence intensity was measured using an LSRFortessa cell analyzer (Becton Dickinson, Franklin Lakes, NJ, USA). The relative levels of costimulatory molecules (CD40, CD80, and CD86) and MHC class II antigen in the CD19^+^ cell population (B cells) and CD11c^+^ cell population (dendritic cells (DCs)) were calculated as follows: median fluorescence intensity (MFI) of the stained cells—MFI of the isotype-matched control cells. The expression levels of costimulatory molecules are presented as specific MFI. Similarly, the levels of IL-10 in the CD4^+^CD25^+^FOXP3^+^ cells or CD19^+^CD1d^+^CD5^+^ cells were calculated as follows: MFI of cells stained with anti-IL-10 antibody—MFI of cells stained with isotype-matched control. In total, 200,000 events were recorded at a rate of 600–800 events per second. Cytometer Setup and Tracking beads (CS&T Research Beads, Becton Dickinson, USA) were used for automated quality assurance and control of machine performance. The analyses were conducted using FlowJo™ software version 10.6.2 (Becton Dickinson, Franklin Lakes, NJ, USA).

### 4.5. Enzyme-Linked Immunosorbent Assay (ELISA)

The blood samples collected from the pregnant mice were centrifuged at 10,000× *g* and 4 °C for 10 min, and the serum samples were stored at −80 °C. The concentrations of TGFβ1, IFN-γ, IL-2, IL-4, and IL-10 were evaluated elevated using the murine Ready-SET-Go! Kit (eBioscience, USA) as previously described [16]. First, the wells of the microplates were incubated with anti-TGFβ1, anti-IFN-γ, anti-IL-2, anti-IL-4, and anti-IL-10 antibodies overnight at 4 °C. Next, the plates were blocked for 1 h at room temperature (RT). The standard concentrations of cytokines or diluted sera (1:5 for TGFβ1 measurements or 1:2 for IFN-γ, IL-2, IL-4, and IL-10 measurements) were added to the wells and incubated overnight at 4 °C. The wells were washed and incubated with biotinylated antibodies for 1 h at RT. The plates were washed thrice and incubated with horseradish peroxidase-conjugated streptavidin for 30 min at RT. Finally, the plates were incubated with 3,3’,5,5’-tetramethylbenzidine substrate for 10 min in the dark at RT. The reaction was terminated with the addition of 50 μL of 1 M H_2_SO_4_. The absorbance at 450 nm (A_450_) was measured using a Wallac 1420 Victor2 microplate reader (PerkinElmer, Waltham, Massachusetts, USA) within 15 min of the endpoint of the protocol.

### 4.6. Statistical Analysis

All statistical analyses were performed using GraphPad Prism 7.0 software (GraphPad Software, San Diego, CA, USA). Normal distribution was assessed using the Shapiro–Wilk normality test, while homoscedasticity was examined using the Brown–Forsythe test. The normally distributed data were analyzed using one-way analysis of variance (parametric), followed by Bonferroni’s multiple comparison post hoc test. The non-normally distributed data were analyzed using the Kruskal–Wallis test (nonparametric), followed by Dunn’s multiple comparison post hoc test. The differences were considered significant at *p* < 0.05.

## Figures and Tables

**Table 1:** Characteristics of designed peptides.

Protein Code	Protein Name	Peptide Sequence and Abbreviation	Impact on Immune System
Q91ZX7.1 in *Mus musculus* Q07954.2 in *Homo sapiens*	prolow-density lipoprotein receptor-related protein 1 isoform X1 (LRP1, CD91)	SGSVVLRNSTTLVMH (SGS)	Scavenger receptor that regulates adaptive immunity and inflammation; the interaction with a variety of ligands and adaptor molecules involved in cell signaling, phagocytosis, and endocytosis [25]
Q09089.1	Outer surface protein A from *Borrelia burgdorferi* (OspA)	LKDFALEGTLAADKT (LKD)	T cells proliferation and secretion of interferon-gamma [26]

## Data Availability

The data presented in this study are available upon request from the corresponding author.
